# Agnogenic massive pulmonary embolism with syncope as initial symptom: A case report and review of the literature

**DOI:** 10.3892/etm.2013.992

**Published:** 2013-03-07

**Authors:** AI-GUI JIANG, HUI-YU LU

**Affiliations:** Department of Respiratory Medicine, Taizhou People’s Hospital, Taizhou, Jiangsu 225300, P.R. China

**Keywords:** pulmonary embolism, interventional therapy, syncope

## Abstract

Pulmonary embolism may escape prompt diagnosis since clinical symptoms and signs are nonspecific. The occurrence of syncope as the sole initial symptom in a previously healthy patient with no predisposing factors to embolism and no hemodynamic instability is extremely rare, which may have been a factor in the delayed diagnosis. We describe a case of agnogenic massive pulmonary embolism with syncope as the initial symptom. A 41-year-old previously healthy female was admitted to the Department of Neurology, Taizhou People’s Hospital in March 2012, for two transitory episodes of syncope during a 5-h period. Following admission, chest computed tomography demonstrated embolism in the right main pulmonary and left inferior pulmonary arteries. Color ultrasonography revealed a dilated right ventricle and right heart overload, severe tricuspid regurgitation and severe pulmonary hypertension. Following the final diagnosis, the patient was successfully treated with interventional mechanical thrombectomy combined with thrombolytic therapy with local and systemic low-dose urokinase. We consider that raised awareness and early diagnosis and treatment were key factors in ensuring a satisfactory prognosis.

## Introduction

Acute massive pulmonary embolism (MPE) is a critical disease associated with high mortality in clinical practice. Approximately 79% of patients have coincident deep vein thrombosis (1). Clinically, the disease mainly manifests as shock and hypotension, and only ∼10% of patients have syncope as the initial symptom (2,3). We report on the diagnosis and treatment of a patient with agnogenic MPE with syncope as the initial symptom.

## Case report

A 41-year-old previously healthy female was admitted to the Department of Neurology, Taizhou People’s Hospital in March 2012, for two transitory episodes of syncope during a 5-h period. The patient had an unhealthy lifestyle of physical inactivity. No urinary and fecal incontinence, general fatigue, chest pain, breathing difficulty, hemoptysis or fever were observed during the course of disease. Following admission, physical examinations revealed a body weight of 75 kg, body height of 159 cm, body temperature of 36.0°C, pulse of 80 bpm, respiratory rate of 23 bpm and blood pressure of 120/60 mmHg. The patient had a slightly haggard expression, no cyanosis of the lips and no jugular vein distention. Bilateral respiratory movements were identical and vocal fremitus was equal. Dullness was heard in the right lower lung on percussion. Breath sounds were diminished and no moist rales were heard. The patient’s heart rhythm was regular, P2>A2 (pulmonary second sound was higher than aortic second heart sound) and there was no edema in the lower extremities.

A complete blood test revealed a white cell count of 11.21×10^9^ cells/l and the percentage of large white blood cells was 57.2%. Biochemical tests revealed 1.4 mmol/l triglycerides, 0.75 mmol/l high-density lipoprotein and 3.61 mmol/l low-density lipoprotein. Blood gas analysis revealed a pH of 7.471, 61.1 mmHg PaO_2_, 23.5 mmHg PaCO_2_ and 18.2 mmol/l HCO_3_^−^ (under the condition of a low flow rate of oxygen inhalation). Chest radiographs revealed pulmonary hilar enlargement and a broadened shadow on the right superior pulmonary artery. An electrocardiogram revealed a flat V1–V3 T wave and magnetic resonance angiography of the head revealed ∼60% luminal stenoses of the right posterior cerebral artery and the left external carotid artery.

**Figure 1:**
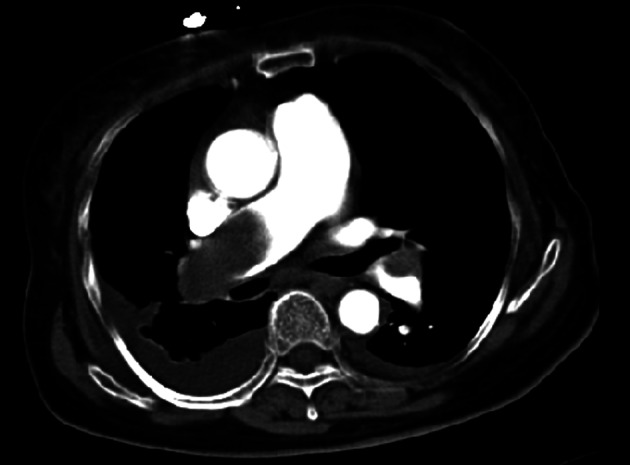
Enhanced chest computed tomography (CT) scan revealing filling defects in the right main pulmonary artery and left lower pulmonary artery branch, as well as bilateral pleural effusion.

**Figure 2:**
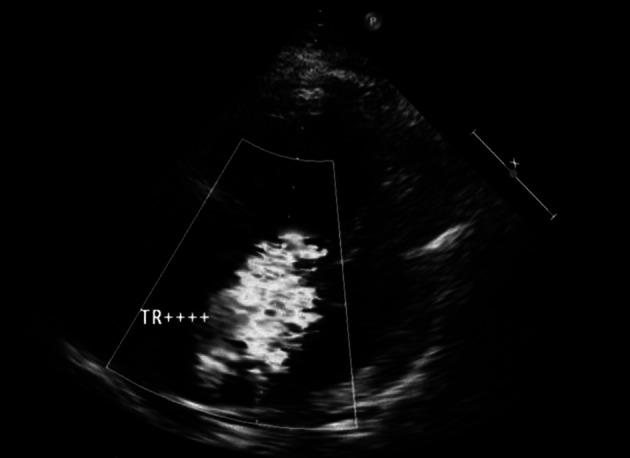
Color ultrasonography of the heart revealing a dilated right ventricle and right heart overload, severe tricuspid regurgitation and severe pulmonary hypertension.

After admission, ‘reflex syncope’ was suspected and the patient was administered oral calcium antagonists (Nimotop 30 mg qd) and intravenous Alprostadil for injection (Alprostadil 10 *μ*g qd) to boost the cerebral circulation, without effect. A further episode of syncope occurred during the 18 h after admission and the patient was transferred to the Department of Respiratory Medicine for a D-dimer assay, which indicated a value of 1,200 *μ*g/l. An enhanced chest computed tomography (CT) scan revealed filling defects in the right main pulmonary and left inferior pulmonary arteries, as well as bilateral pleural effusion (Fig. 1). Color ultrasonography of the heart revealed a dilated right ventricle and right heart overload, severe tricuspid regurgitation and severe pulmonary hypertension and the systolic pulmonary arterial pressure was 130 mmHg (Fig. 2). The patient was finally diagnosed with MPE.

Following confirmation, the patient underwent interventional mechanical thrombectomy combined with local and systematic thrombolytic therapy with low-dose urokinase.

**Figure 3:**
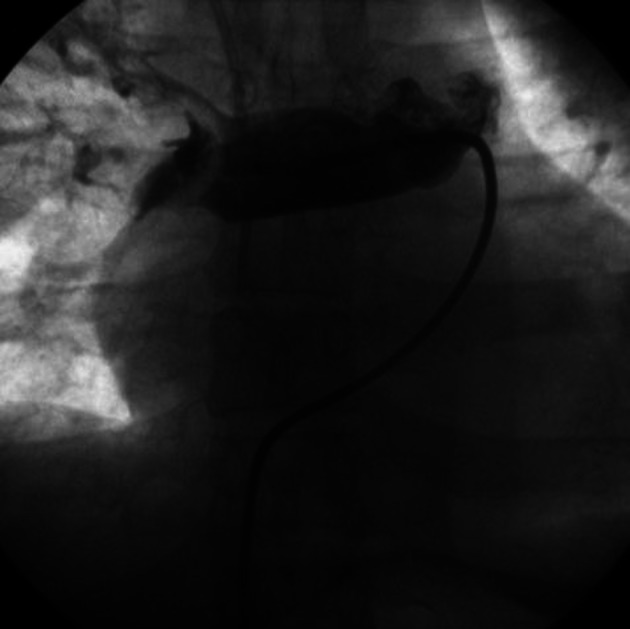
Digital subtraction angiography of pulmonary artery confirming the filling defect in the right pulmonary artery.

**Figure 4:**
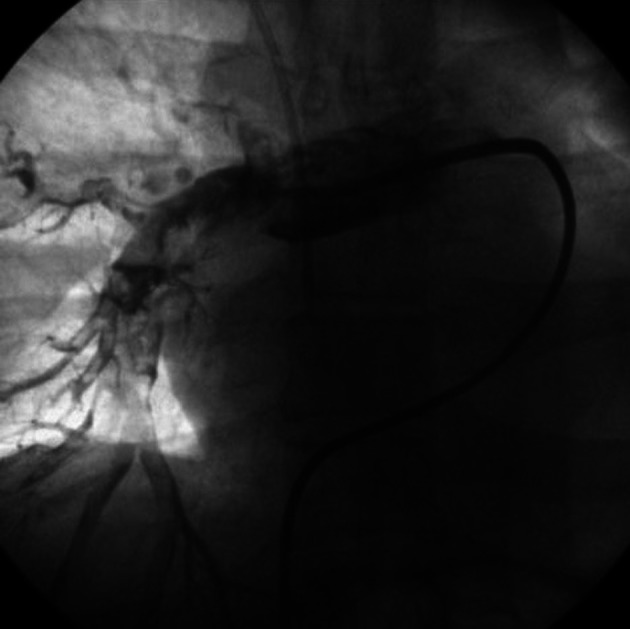
Digital subtraction angiography demonstrating an improved filling defect in the pulmonary artery following interventional mechanical embolus disintegration.

Following the above therapies, digital subtraction angiography (DSA) of the deep veins of the lower limbs and the inferior vena cava demonstrated unobstructed blood flow, with no apparent thrombosis. A 4–5F double J tube was inserted through the right femoral vein to the main pulmonary artery for DSA of the pulmonary artery, to confirm the filling defect in the right main pulmonary artery (Fig. 3). An exchange guide wire was then inserted to coordinate with the tube for twists and drags to disintegrate the embolus. Following disintegration of the embolus, 500,000 units urokinase were injected into the tube for thrombolysis over 30 min. Subsequent DSA of the pulmonary artery indicated an improvement in the filling defect compared with before treatment (Fig. 4). The patient’s condition was significantly alleviated and the anoxia was reduced. A blood gas assay performed 2 h after surgery indicated a pH of 7.51, 72 mmHg PaO_2_, 29 mmHg PaCO_2_ and 23.1 mmol/l HCO_3_^−^ (under the condition of a low flow rate of oxygen inhalation).

A postoperative intravenous drip of low-dose urokinase (200,000 units) was initiated and the patient was also treated with 5,000 units low-molecular-weight heparin, administered subcutaneously once every 12 h for three consecutive days. Warfarin (2.5 mg) was administered orally once every 12 h on a daily basis. The prothrombin time (PT) and international normalized ratio (INR) were monitored; when the PT and INR were twice and 2.5 times their respective normal levels, warfarin therapy was administered singly, plus anti-infective, supportive and oxygen therapies.

**Figure 5:**
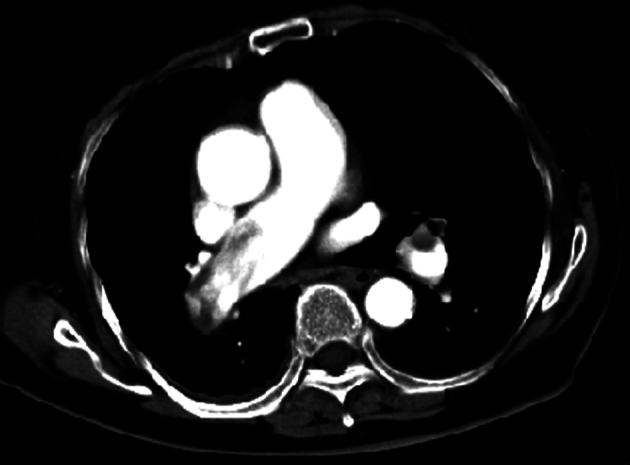
Enhanced chest computed tomography (CT) scan revealing an improved pulmonary embolism in the right main pulmonary artery and left inferior pulmonary artery, as well as disappearance of the hydrothorax.

Three days after surgery, the patient demonstrated a distinctly improved mental condition with no further syncopal attacks. An enhanced chest CT scan 10 days after surgery revealed evident improvement of the thrombosis in the right main pulmonary artery and left inferior pulmonary artery branch, as well as disappearance of the pleural effusion, compared with the previous chest CT scan (Fig. 5). Color ultrasonography revealed a significant decrease of pulmonary artery pressure and right heart load; the systolic pulmonary arterial pressure was 71 mmHg. The patient was discharged from hospital 26 days after admission, with continued daily administration of 2.5 mg warfarin. Based on the monitoring of PT and INR, the doctor suggested discontinuation of warfarin 3 months after hospital discharge. The study was approved by the Ethics Committee of Taizhou People’s Hospital, Jiangsu, China and according to the Declaration of Helsinki. Written informed consent was obtained from the patient.

## Discussion

MPE refers to an embolism of the main pulmonary trunk and is associated with high mortality (4,5). The main clinical manifestations usually include shock and hypotension as the initial symptoms, commonly with comorbid right heart dysfunction. The embolus mainly arises from the deep vein system of the lower limbs or right heart system, or from the deep veins of the upper limbs (6). MPE caused by uncertain risk factors is rarely reported. The patient presented in this study was otherwise healthy and had no predisposing factors to embolism, with normal blood lipid levels. There were no distinct signs of embolism on DSA of the deep lower limb veins or inferior vena cava or in B-ultrasound examination of the upper limbs. A complete enhanced CT scan of the abdomen revealed no abnormalities. We consider that the possible causes may be: i) long periods of time sitting everyday and an unhealthy lifestyle of physical inactivity resulted in a higher risk of developing a life-threatening blood clot in the lungs than that in active women (7). ii) Although ∼79% of patients who present with pulmonary embolism have evidence of deep venous thrombosis in their legs, if deep venous thrombosis is not detected in such patients, it is likely that the whole thrombus has already detached and embolised (1). Nonetheless, this patient represented a rare clinical presentation, which is often neglected by clinicians as a result of missed and incorrect diagnoses.

Syncope as the sole initial symptom has been reported in MPE patients with comorbid hemodynamic disturbances (3,8,9). There are several possible causes: i) acute right heart failure and damaged pulmonary blood perfusion causing decreased filling of the left ventricle, with resulting hypotension, bradycardia and cerebral circulation disturbance (10). ii) Reflex syncope caused by bradycardia due to vagal stimulation and by peripheral vascular distention due to suppression of sympathetic nerves (11) and iii) syncope caused by an atrioventricular block induced by MPE (12). In the present case, the patient’s clinical manifestation was transitory syncope closely associated with activities. Considering its duration of onset, modes of relief and basic conditions, the patient may easily have been misdiagnosed with syncope due to a nervous system disease, thus missing the precious opportunity for treatment. When the cause of syncope remains unknown, timely D-dimer assay and blood gas analysis effectively reduces the risk of a missed or inaccurate diagnosis.

MPE combined with hemodynamic disturbance is an absolute indication of thrombolysis; although thrombolytic therapy via a peripheral vein has a delayed effect and a high risk of hemorrhage. Certain patients are unable to benefit from this treatment due to contraindications to thrombolysis. Surgical thrombectomy of the pulmonary artery is also associated with high mortality and disability risks (13). However, advances in interventional therapies and instruments mean that the interventional treatment of acute MPE has achieved satisfactory effects and has received increasing attention (14). The usual methods include local pulmonary thrombolysis via a catheter, mechanical embolus disintegration and thrombectomy via a catheter.

Compared with venous thrombolysis, local pulmonary artery thrombolysis via a catheter elevates the focal drug concentration and reduces the required dosage of thrombolytic medications, thus enhancing the therapeutic effects and reducing the incidence of hemorrhagic complications. The procedure of mechanical embolus disintegration and thrombectomy by a catheter permits the clearance of embolus fragments and entry into the distal pulmonary artery, realizes the unblocking of the clogged central pulmonary artery, improves pulmonary perfusion, reduces pulmonary artery pressure and improves right ventricular function, consequently resulting in an increased clinical treatment success rate.

In conclusion, the occurrence of syncope as the sole initial symptom in a previously healthy patient with no predisposing factors to embolism and no hemodynamic instability following admission is extremely rare, which may have been a factor in the delayed diagnosis of pulmonary thromboembolism. In this situation, the raised awareness of diagnosis and knowledge concerning the clinical presentation of pulmonary thromboembolism are key factors in ensuring an immediate diagnosis and adequate intervention.

## References

[1] Tapson V (2008). Acute pulmonary embolism. N Engl J Med.

[2] Calvo-Romero JM, Pérez-Miranda M, Bureo-Dacal P (2004). Syncope in acute pulmonary embolism. Eur J Emerg Med.

[3] Castelli R, Tarsia P, Tantardini C, Pantaleo G, Guariglia A, Porro F (2003). Syncope in patients with pulmonary embolism: comparison between patients with syncope as the presenting symptom of pulmonary embolism and patients with pulmonary embolism without syncope. Vasc Med.

[4] Wood KE (2002). Major pulmonary embolism: review of a pathophysiologic approach to the golden hour of hemodynamically significant pulmonary embolism. Chest.

[5] Schoepf UJ, Kucher N, Kipfmueller F, Quiroz R, Costello P, Goldhaber SZ (2004). Right ventricular enlargement on chest computed tomography: a predictor of early death in acute pulmonary embolism. Circulation.

[6] Basat HC, Kalem M, Binnet MS, Demirtas M (2011). Pulmonary thromboembolism after surgical treatment of ulnar pseudoarthrosis: a case report. Acta Orthop Traumatol Turc.

[7] Kabrhel C, Varraso R, Goldhaber SZ, Rimm E, Camargo CA (2011). Physical inactivity and idiopathic pulmonary embolism in women: prospective study. BMJ.

[8] Mahboobi SK, Shohat EZ (2005). Syncope: an unusual presentation of acute pulmonary embolism. South Med J.

[9] Sarasin FP, Louis-Simonet M, Carballo D, Slama S, Rajeswaran A, Metzger JT (2001). Prospective evaluation of patients with syncope: a population-based study. Am J Med.

[10] Gossage JR (2002). Early intervention in massive pulmonary embolism. A guide to diagnosis and triage for the critical first hour. Postgrad Med.

[11] Eldadah ZA, Najjar SS, Ziegelstein RC (2000). A patient with syncope, only ‘vagally’ related to the heart. Chest.

[12] Elias J, Kuniyoshi R, Moulin B (2004). Syncope and complete atrioventricular block related to pulmonary thromboembolism. Arq Bras Cardiol.

[13] Pulido-Zamudio T, Reyes-Fuentes LF, Beltrán-Gámez M (2012). Management of acute pulmonary thromboembolism. Arch Cardiol Mex.

[14] Skaf E, Beemath A, Siddiqui T, Janjua M, Patel NR, Stein PD (2007). Catheter-tip embolectomy in the management of acute massive pulmonary embolism. Am J Cardiol.

